# The association between renal sinus fat area and the progression-free survival in Chinese non-metastatic clear-cell renal cell carcinoma patients

**DOI:** 10.18632/oncotarget.19012

**Published:** 2017-07-05

**Authors:** Haichao Huang, Shi Chen, Wei Yu, Zirong Ye, Wei Li, Jinchun Xing, Xiurong Wu

**Affiliations:** ^1^ Department of Urology, The First Affiliated Hospital of Xiamen University, Siming District, Xiamen, Fujian 361003, China; ^2^ Department of Radiology, The First Affiliated Hospital of Xiamen University, Siming District, Xiamen, Fujian 361003, China; ^3^ Department of Urology, Peking University First Hospital and Institute of Urology, Peking University, National Urological Cancer Center, Xicheng District, Beijing 100034, China; ^4^ Key Laboratory of Health Technology Assessment of Fujian Province University, School of Public Health, Xiamen University, Xiamen, Fujian 361005, China

**Keywords:** renal sinus fat, progression, survival, non-metastatic, renal cell carcinoma

## Abstract

In this retrospective study, we evaluated the association between renal sinus fat area (RSFA) and survival in 268 Chinese non-metastatic clear-cell renal cell carcinoma (ccRCC) patients. Patients with high RSFA exhibited better progression-free survival than those with low RSFA in both univariable (HR: 0.240; 95% CI: 0.119–0.482; *p* < 0.001) and multivariable (HR: 0.432; 95% CI: 0.369–2.749; *p* = 0.027) analyses. A propensity-score matched (PSM) analysis using Kaplan-Meier curves confirmed our findings (log-rank test; *p* = 0.028). Based on the multivariable analysis, we constructed a prognostic nomogram with 4 factors, namely, RSFA, Fuhrman grade, AJCC stage and sarcomatoid component. The c-index values for the Leibovich scoring system and the nomogram were 0.762 (95%CI, 0.688–0.835) and 0.823 (95%CI, 0.759–0.888), respectively. These findings demonstrate that high RSFA is associated with better progression-free survival in non-metastatic ccRCC.

## INTRODUCTION

Renal cell carcinoma (RCC) represents 2–3% of all human malignances and the most common histological subtype is clear-cell renal cell carcinoma (ccRCC) [1–2]. Many post-operative clinicopathological features are associated with overall survival of RCC patients and several predictive score models have been established to improve the risk stratification [3–5]. Metabolic syndrome (MS) is one of the pre-operative parameters that have been recently reported that predict survival outcomes of RCC patients. Two major features of MS, namely, visceral obesity and hypertension have been established as etiological factors of RCC [6–7]. However, contradictory results have been reported regarding association between visceral fat area (VFA) and the prognosis of RCC [8–13]. Recently, renal sinus fat accumulation (RSFA) or ectopic adipose tissue deposits have been associated with many MS features including hypertension [14–15]. However, the role of RSFA in RCC is unknown. Therefore, we studied the association between renal sinus fat area (RSFA) and the progression-free survival (PFS) of ccRCC. In addition, we constructed a nomogram to predict the PFS of non-metastatic ccRCC.

## RESULTS

### Baseline characteristics

We included 268 patients with pathologically confirmed ccRCC (126 located in the left kidney; 142 in the right) that underwent partial (*n =* 82) and radical (*n =* 186) nephrectomy in the present study. Patients with high RSFA values correlated with high SFA, VFA and BMI value and showed a high proportion of smaller tumor size, lower AJCC stage and lower fuhrman grade. Patients with low RSFA were more likely to have renal sinus invasion. We performed a one-to-one PSM analysis because the two study cohorts were not fully comparable. In the matched cohorts (*n =* 162; 81 pairs), the variables were fully comparable without statistical significance (Table 1). In addition, 43 patients were diagnosed as DM cases. The distribution of pre-operative HbA1c values (ranging from 5.8% to 10.5%) were as follows: 2 above 8%, 12 between 7–8%, 9 between 6.5–7% and 20 cases less than 6.5%. Further evaluation of the tumor grade was performed according to the WHO/ISUP grading system. There were 67, 128, 55 and 18 patients that were identified as grade 1, grade 2, grade 3 and grade 4, respectively. Patients with high RSFA values also correlated with lower WHO/ISUP grade than those with low RSFA values (*p* = 0.007). We identified 39, 71, 19 and 5 patients as grade 1, grade 2, grade 3 and grade 4, in the high RSFA group, respectively. We also identified 28, 57, 36 and 13 patients as grade 1, grade 2, grade 3 and grade 4 in the low RSFA group, respectively.

**Table 1:** Descriptive clinicopathologic characteristics of patients with non-metastatic renal cell carcinoma before (*n* = 268) and after (*n* = 162) propensity-score matching

	Original unmatched cohorts (*n* = 268)			Propensity-score matched cohorts (*n* = 162)		
	Low RSFA, *n* (%)	High RSFA, *n* (%)	*P*	Low RSFA, *n* (%)	High RSFA, *n* (%)	*P*
Location side			0.807			0.752
left	62 (46.3)	64 (47.8)		38 (46.9)	36 (44.4)	
right	72 (53.7)	70 (52.2)		43 (53.1)	45 (55.6)	
Age (years)			0.306			0.870
less than 60	91 (67.9)	83 (61.9)		51 (63.0)	52 (64.2)	
60 or greater	43 (32.1)	51 (38.1)		30 (37.0)	29 (35.8)	
Gender			0.603			1.000
male	88 (65.7)	92 (68.7)		53 (65.4)	53 (65.4)	
female	46 (34.3)	42 (31.3)		28 (34.6)	28 (34.6)	
SFA value			0.003			0.752
high	55 (41.0)	79 (59.0)		44 (54.3)	46 (56.8)	
low	79 (59.0)	55 (41.0)		37 (45.7)	35 (43.2)	
VFA value			0.000			0.753
high	50 (37.3)	84 (62.7)		41 (50.6)	39 (48.1)	
low	84 (62.7)	50 (37.3)		40 (49.4)	42 (51.9)	
Hypertension			0.062			0.861
absent	101 (75.4)	87 (64.9)		58 (71.6)	59 (72.8)	
present	33 (34.6)	47 (35.1)		23 (28.4)	22 (37.2)	
DM			0.013			0.375
absent	120 (89.6)	105 (78.4)		68 (84.0)	71 (87.7)	
present	14 (10.4)	29 (21.6)		13 (16.0)	9 (12.3)	
BMI			0.011			0.870
less than 25	104 (77.6)	81 (60.4)		52 (64.2)	51 (63.0)	
25 or greater	30 (22.4)	53 (39.6)		29 (35.8)	30 (37.0)	
Tumor size (cm)			0.018			0.311
less than 5	68 (50.7)	88 (65.7)		51 (63.0)	44 (54.3)	
5 or greater	54 (40.3)	42 (31.3)		24 (29.6)	33 (40.7)	
10 or greater	12 (9.0)	4 (3.0)		6 (7.4)	4 (35.0)	
Fuhrman grade			0.010			0.369
grade 1	28 (20.9)	36 (26.9)		19 (23.5)	21 (25.9)	
grade 2	59 (44.0)	75 (56.0)		41 (50.6)	43 (53.1)	
grade 3	41 (30.6)	21 (15.7)		21 (25.9)	15 (18.5)	
grade 4	6 (4.5)	2 (1.4)		0 (0)	2 (2.5)	
AJCC stage			0.001			0.289
stage I	87 (64.9)	113 (84.3)		58 (71.6)	64 (79.0)	
stage II	24 (17.9)	11 (8.2)		15 (18.5)	8 (9.9)	
stage III	23 (17.2)	10 (7.5)		8 (9.9)	9 (11.1)	
T stage			0.002			0.506
T1	88 (65.7)	113 (84.3)		58 (71.6)	64 (79.0)	
T2	24 (17.9)	13 (9.7)		15 (18.5)	10 (12.3)	
T3	22 (16.4)	8 (6.0)		8 (9.9)	7 (8.7)	
LNM			0.702			0.613
absent	130 (97.0)	131 (97.8)		80 (98.8)	78 (96.3)	
present	4 (3.0)	3 (2.2)		1 (1.2)	3 (2.7)	
RSF invasion			0.001			0.613
absent	121 (90.3)	133 (99.3)		78 (96.3)	80 (98.8)	
present	13 (9.7)	1 (0.7)		3 (2.7)	1 (1.2)	
Histological necrosis			1.000			0.588
absent	99 (73.9)	99 (73.9)		59 (72.8)	62 (76.5)	
present	35 (26.1)	35 (26.1)		22 (27.2)	19 (23.5)	
Sarcomatoid differentiation			0.055			0.316
absent	128 (95.5)	133 (99.3)		81 (100.0)	80 (98.8)	
present	6 (4.5)	1 (0.7)		0 (0)	1 (1.2)	

SFA = subcutaneous fat area; VFA = visceral fat area; RSFA = renal sinus fat area; DM = diabetes mellitus; BMI = body mass index; AJCC = American Joint Committee on Cancer; LNM = lymph node metastasis.

### Survival outcomes

**Figure 1:**
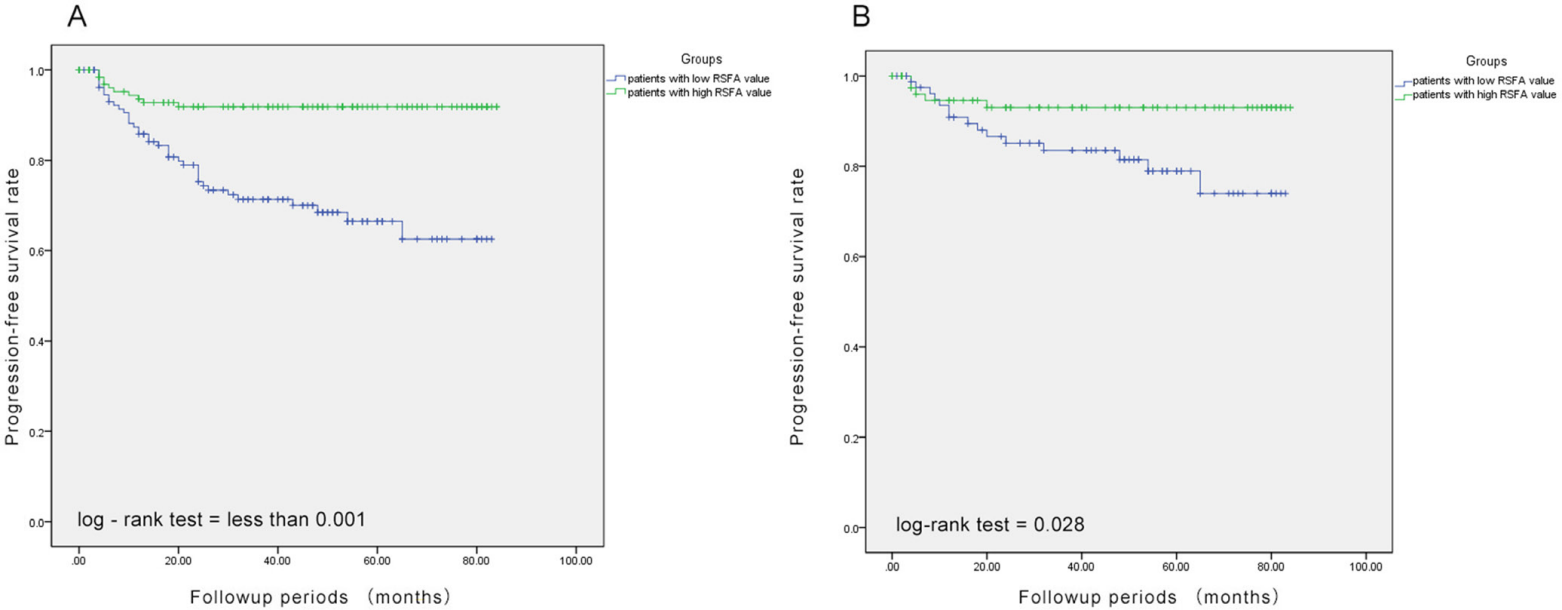
Association between renal sinus fat area and progression-free survival (**A**) Patients with high renal sinus fat area (green line) show better progression-free survival than those with low renal sinus fat area (blue line). (**B**) Propensity-score matching analysis demonstrates that patients with high renal sinus fat area show better progression-free survival than those with low renal sinus fat area.

The median follow-up of the 268 patients was 38 months with 48 patients (16 with AJCC stage I, 15 with stage II, and 17 with stage III) experiencing progression (distant metastasis or local recurrence) after surgery. Patients with high RSFA showed longer progression-free survival (PFS) than those with low RSFA (*p* < 0.001; Figure 1A). The 3-year PFS was 71.3% and 91.8% for patients with low and high RSFA. In the univariable Cox analysis, high SFA, VFA, and RSFA values, lower Fuhrman and WHO/ISUP grades, lower AJCC stage, smaller tumor size, absence of sarcomatoid component and renal sinus invasion were associated with higher PFS. However, multivariable analysis demonstrated that high RSFA, lower Fuhrman and WHO/ISUP grades, lower AJCC stage and absence of sarcomatoid component were associated with high PFS (Tables 2 and 3).

**Table 2:** Univariate and multivariate Cox regression analyses for prediction of progression-free survival in 268 non-metastatic cc-RCC patients treated with nephrectomy (Tumor grade was identified according to the Fuhrman grading system in the multivariable system)

Univariate analyses	HR	95% CI	*P* value
SFA (high)	0.461	0.253–0.841	0.012
VFA (high)	0.466	0.201–1.081	0.075
RSFA (high)	0.240	0.119–0.482	< 0.001
BMI (overweight/obese)	0.728	0.385–1.377	0.328
Gender (male)	1.186	0.637–2.211	0.590
Age (≥ 60 yr)	0.758	0.406–1.412	0.382
Fuhrman grade			< 0.001
grade 2 vs. grade 1	2.850	0.835–9.726	0.094
grade 3 vs. grade 1	8.935	2.642–30.211	< 0.001
grade 4 vs. grade 1	27.042	7.247–100.902	< 0.001
WHO/ISUP grade			< 0.001
grade 2 vs. grade 1	4.533	1.042 – 19.717	0.044
grade 3 vs. grade 1	13.539	3.139 – 58.395	< 0.001
grade 4 vs. grade 1	43.611	9.686–196.368	< 0.001
Tumor size			< 0.001
middle vs. small	4.496	2.246–9.002	< 0.001
large vs. small	10.014	4.017–24.961	< 0.001
Histological necrosis (present)	1.303	0.707–2.399	0.396
AJCC stage			< 0.001
stage 2 vs. stage 1	6.123	3.025–12.391	< 0.001
stage 3 vs. stage 1	9.160	4.613–18.186	< 0.001
T stage			< 0.001
T2 vs. T1	5.928	2.993–11.742	< 0.001
T3 vs. T1	8.092	4.031–16.241	< 0.001
LNM (present)	7.103	2.783–18.126	< 0.001
RSF invasion (present)	5.924	2.749–12.768	< 0.001
Hypertension (present)	1.001	0.543–1.843	0.999
Diabetes (present)	1.074	0.502–2.295	0.854
Sarcomatoid differentiation (present)	19.110	8.264–44.189	< 0.001
Tumor location (right)	0.567	0.318–1.012	0.055

Multivariate analyses	HR	95% CI	*P*
SFA (high)	0.818	0.412–1.625	0.566
VFA (high)	0.569	0.287–1.127	0.106
RSFA (high)*	0.432	0.206–0.907	0.027
RSF invasion (present)	1.007	0.369–2.749	0.988
Fuhrman grade*			0.007
grade 2 vs. grade 1	5.396	1.285–22.657	0.021
grade 3 vs. grade 1	7.015	1.588–30.987	0.010
grade 4 vs. grade 1	13.554	3.109–59.091	0.001
Tumor size			0.688
middle vs. small	1.433	0.578–3.551	0.437
large vs. small	1.640	0.483–5.565	0.427
AJCC stage*			0.042
stage 2 vs. stage 1	2.556	1.059–6.170	0.037
stage 3 vs. stage 1	3.502	1.224–10.018	0.019
Sarcomatoid differentiation (present)*	9.830	2.588–37.343	0.001
Tumor location (right)	0.852	0.445–1.633	0.630

cc-RCC = clear-cell renal cell carcinoma; SFA = subcutaneous fat area; VFA = visceral fat area; RSFA = renal sinus fat area; BMI = body mass index; AJCC = American Joint Committee on Cancer; LNM = lymph node metastasis. *= *p* less than 0.05 in multivariable analysis.

**Table 3:** Multivariate Cox regression analyses for prediction of progression-free survival in 268 non-metastatic cc-RCC patients treated with nephrectomy (Tumor grade was identified according to the WHO/ISUP grading system)

Multivariate analyses	HR	95% CI	*P*
SFA (high)	0.862	0.436–1.706	0.670
VFA (high)	0.569	0.286–1.131	0.108
RSFA (high)*	0.465	0.221–0.975	0.043
RSF invasion (present)	1.012	0.372–2.753	0.982
WHO/ISUP grade*			0.009
grade 2 vs. grade 1	5.244	1.157–23.763	0.032
grade 3 vs. grade 1	6.494	1.358–31.054	0.019
grade 4 vs. grade 1	13.554	14.737–72.395	0.001
Tumor size			0.689
middle vs. small	1.442	0.592–3.509	0.420
large vs. small	1.604	0.471–5.457	0.450
AJCC stage*			0.031
stage 2 vs. stage 1	2.303	0.940–5.643	0.068
stage 3 vs. stage 1	3.987	1.411–11.266	0.009
Sarcomatoid differentiation (present)*	5.235	1.539–17.802	0.008
Tumor location (right)	0.925	0.481–1.779	0.816

cc-RCC = clear-cell renal cell carcinoma; SFA = subcutaneous fat area; VFA = visceral fat area; RSFA = renal sinus fat area; AJCC = American Joint Committee on Cancer. *= *p* less than 0.05 in multivariable analysis.

**Figure 2:**
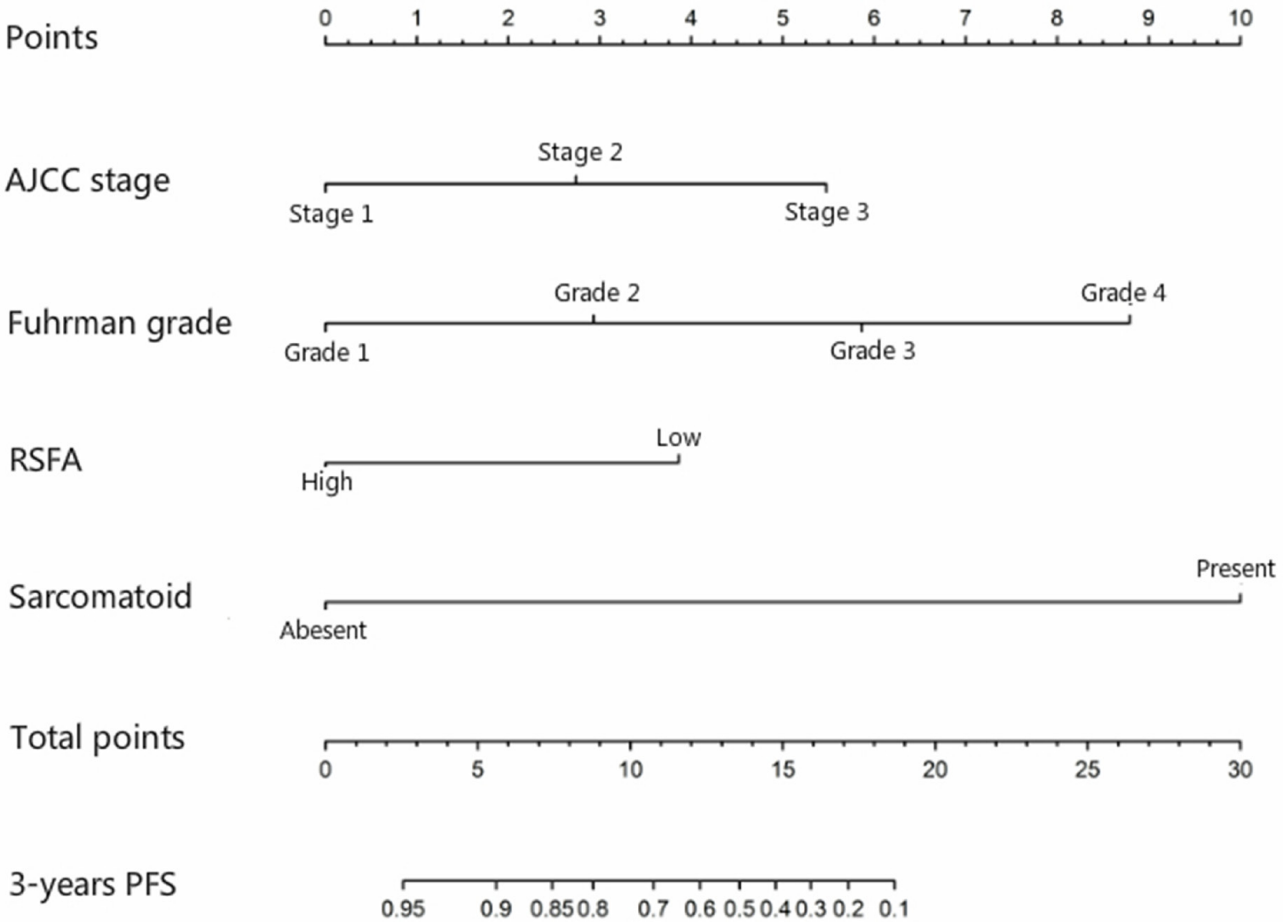
Nomogram for predicting 3-year progression-free survival of non-metastatic clear-cell renal cell carcinoma using renal sinus fat area (RSFA), AJCC stage, Fuhrman grade and sarcomatoid differentiation parameters

**Figure 3:**
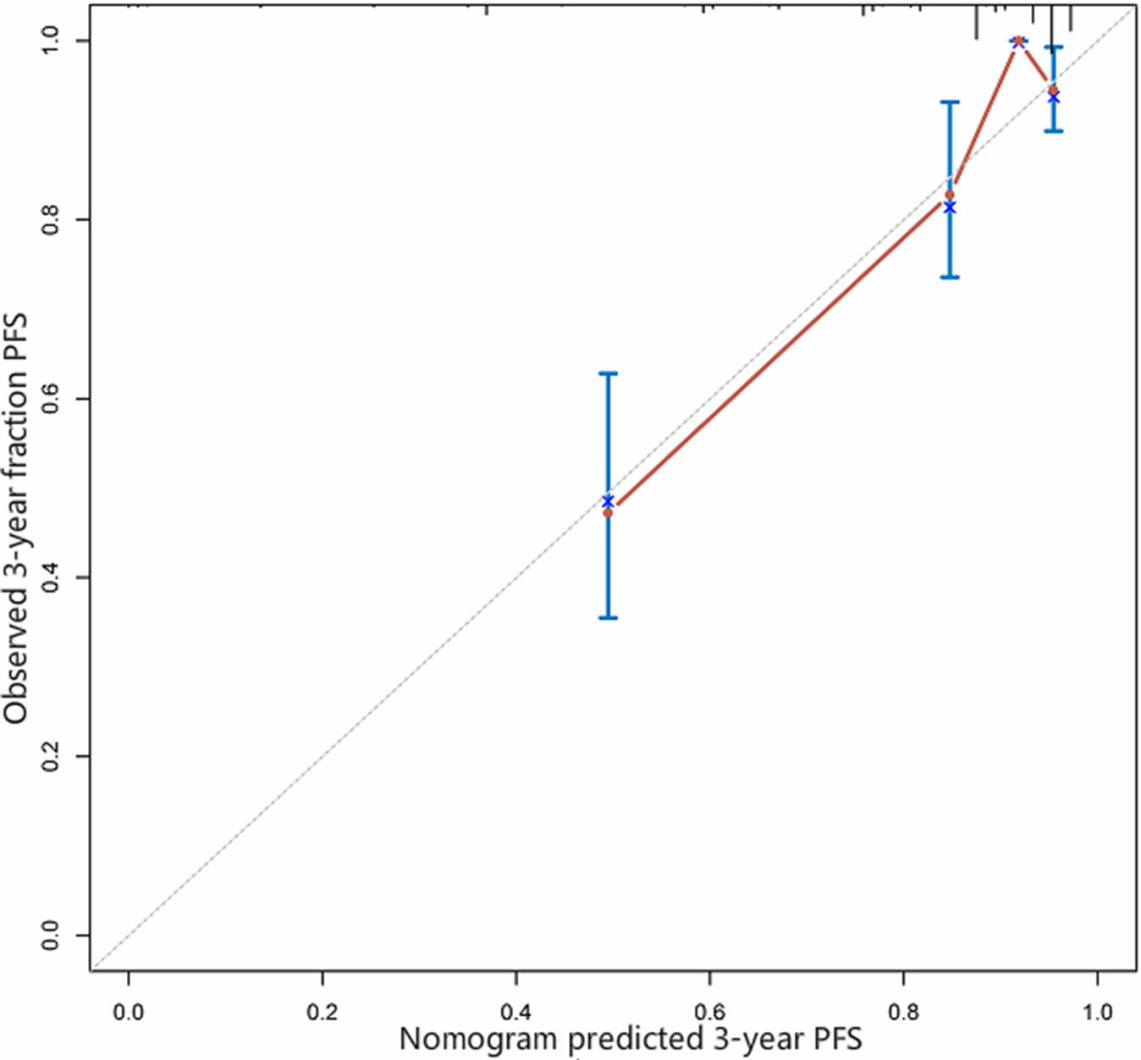
Calibration curve for predicted and observed 3-year progression-free survival

Further, our analysis demonstrated that larger RSFA, lower Fuhrman and WHO/ISUP grades, lower AJCC stage and the presence of sarcomatoid component were independent predictive factors of the PFS of non-metastatic ccRCC. Leibovich scoring system used Fuhrman grade as one of the predictors. Thus, we established a prognostic nomogram with factors such as Fuhrman grade, RSFA, AJCC stage and the presence of sarcomatoid component (Figure 2). The calibration curves demonstrated good consistency in bootstrap analysis between the calculated and actual 3-year PFS (Figure 3). The c-index values of Leibovich scoring system and our new nomogram were 0.762 (95%CI, 0.688–0.835) and 0.823 (95%CI, 0.759–0.888), respectively. In matched cohorts, 20 patients experienced tumor progression with the 3-year PFS values for patients with low and high RSFA being 83.5% and 93.0%, respectively. Further, survival analysis revealed that patients with larger RSFA value were associated with better PFS compared to patients with low RSFA (*p* = 0.028; Figure 1B).

## DISCUSSION

Metabolic syndrome (MS) is primarily associated with development of cardiovarcular disease, hypertension and type 2 diabetes. Recently, its role in many cancers including RCC has been recognized. Among the many features of MS, obesity and hypertension have been identified as etiological factors worldwide [6–7]. Obesity is associated with the prevalence of a variety of cancers, including RCC (especially ccRCC). It is postulated that adipose tissue derived hormones in obesity regulate angiogenesis, epithelial-mesenchymal transition, and inflammation during tumor progression [16].

However, contradictory results have been reported regarding the association of visceral adipose tissue accumulation with RCC progression. Lee and colleagues reported that larger visceral fat area (greater than 50% in both sexes) was associated with longer survival of advanced RCC patients of Korean origin [11]. Likewise, Kaneko and colleagues reported similar findings in their study [10]. In contrast, Mano and colleagues recently reported that neither SFA nor VFA were associated with the survival of non-metastatic ccRCC in a western population [9]. Furthermore, Ladoire and colleagues suggested that high VFA was a predictive biomarker for lower survival in metastatic RCC patients that were given first-line anti-angiogenic drugs [12]. In our study, high SFA and VFA values showed better PFS compared to corresponding low values in the univariable analysis. However, they were not associated according to multivariable analysis. Therefore, the role of VFA and SFA in the development and progression of ccRCC remains unclear and further studies with larger cohorts are necessary.

Epidemiologic studies have demonstrated that hypertension is one of the strongest risk factors for RCC in western populations [17–18]. A recent investigation in Chinese population demonstrated that hypertension played a significant role in the etiology of RCC [19]. Also, patients with higher renal sinus fat accumulation (measured as area or volume) were associated with increased risk of hypertension [14–15]. Therefore, we assessed the association between RSFA values and the PFS of ccRCC by evaluating the CT scans. In our study, patients with high RSFA showed better PFS than those with low RSFA in both univariable and multivariable Cox analysis. Further, we used the PSM analysis to confirm our findings because it reduces bias due to confounding baseline factors [20]. PSM analysis also showed a strong relationship between high RSFA and better RCC survival. In addition, the nomogram including RSFA, AJCC stage, Fuhrman grade, sarcomatoid differentiation parameters demonstrated a more accurate prognosis for PFS than the Leibovich scoring system, which is the most commonly used predictive model to determine PFS of ccRCC.

The major branches of the renal artery and vein along with the major and minor calices of the collecting system, ureters and lymph vessels are located within the renal sinus. Therefore, we hypothesized that excess fat tissue deposits in the renal sinus may act as a physical barrier and prevent tumor cells from migrating to distant organs through veins and lymph vessels. Moreover, Zi and colleagues demonstrated that among perineoplasm, renal sinus, and adipose tissue conditioned media, only perineoplasm conditioned medium enhanced the migration of ccRCC cells (CaKi-2 cells) due to enhanced WNT signaling [21]. Therefore, we hypothesized that the association of RSFA with PFS of ccRCC might be due to physical factors and not due to biological factors. Among the 14 patients that showed pathological renal sinus invasion, 13 belonged to the low RSFA group, while the remaining 1 belonged to the high RSFA group. Since we had only 1 patient with high RSFA and renal sinus invasion, we were unable to evaluate the differences in PFS between high and low RSFA patients with renal sinus invasion. Therefore, future studies are necessary to evaluate if high renal sinus fat accumulation acts as a physical barrier and prevents distal metastasis. It is also worth noting that patients with aggressive tumors experienced considerable weight loss. However, it’s unclear if weight loss was a consequence of cachexia. Therefore, further studies are necessary to elucidate the mechanisms mediating renal sinus fat accumulation and its association with RCC prognosis.

CT scans are routinely used for standard evaluation for RCC patients. Therefore images for assessment of RSFA are readily available. Therefore, the status of pre-operative RSFA can be easily evaluated and used in the clinic. However, standard cut-off values to distinguish high and low RSFA have not yet been determined. In addition, reference values of RSFA based on ethnicity are also not available. Likewise, reference value limits for VFA to predict RCC prognosis are not available. In previous studies on association between VFA and RCC prognosis, patients were divided into obese and non-obese groups based on the median value. Further, since adipose tissue distribution is different between males and females, use of sex-specific median value was recommended [11]. In our study, we sub-divided the patient cohorts based on sex-specific median values. Also, we took the side of the kidney into consideration in regard to RSFA, tumor location and their association with PFS of ccRCC patients. However, we were limited by the lack of standard normal reference value of RSFA in regard to gender and race. Mano and colleagues reported the median values of sex-adjusted VFA as 218.62 cm^2^ and 156.49 cm^2^ for male and female, respectively in a western population with non-metastatic ccRCC [9]. However, the median values of VFA in our study were 123.11 cm^2^ and 80.92 cm^2^ for males and females, respectively. Lee and colleagues reported sex-specific median values of VFA as 117.07 cm^2^ and 79.28 cm^2^ for male and females, respectively, which were similar to our reported values [11] Foster and colleagues evaluated the RSFA of 92 participants with 49% women (100% right kidney) and reported a median RSFA value of 0.43 cm^2^ [15]. However, we reported larger RSFA values (1.28 cm^2^ for males and 0.59 cm^2^ for females) than those reported by Foster and colleagues (0.43 cm^2^).

The limitations of our investigation include the inherent bias due to the retrospective nature of our study, relative small sample size, and short follow-up periods. Because of a relatively shorter follow-up period and a small number of deaths in that period, we were unable to evaluate the association between RSFA and overall survival and cancer-specific survival. Furthermore, we only evaluated non-metastatic ccRCC. Therefore, predictive value of RSFA in other histological types of RCC or metastatic RCC is unknown. Also, the lack of clear-cut mechanistic details and standard RSFA cut-off values limit the utility of RSFA in clinical practice.

In conclusion, our study demonstrates that high RSFA is associated with increased progression-free survival of ccRCC in Chinese patients.

## MATERIALS AND METHODS

### Patient enrollment

We retrospectively enrolled 268 patients that underwent nephrectomy at our center between December 2009 and December 2015 and were post-operatively diagnosed as non-metastatic ccRCC. This study was approved by the institutional ethics committee of The First Affiliated Hospital of Xiamen University. A waiver of written informed consent was granted by the ethics committee for this retrospective analysis. Patient records/information was treated anonymously for this analysis.

Patients were excluded from the analyses if (1) they were operated in our center but had received CT scans in other hospitals before operation and therefore digital CT images were not available for analysis; (2) they did not undergo operation (partial or radical nephrectomy) as the main treatment; and (3) they had surgery elsewhere. All surgical procedures were performed by the director (JCX) of our center.

### Clinicopathological parameters

We assessed clinicopathological factors such as gender, age at the diagnosis (> 60 y vs. < 60 y), body mass index (BMI, ≥ 25 kg/m^2^ vs. < 25 kg/m^2^), kidney side where tumors were located (right vs. left), nephrectomy type (partial vs. radical), VFA and subcutaneous fat area (SFA) at the level of the umbilicus, RSFA, pathological stage based on the 2010 American Joint Committee on Cancer (AJCC), tumor size (< 5 cm, 5–10 cm vs. > 10 cm), Fuhrman grading (grades 1–4), sarcomatoid component, pathological renal sinus invasion, hypertension and diabetes mellitus (DM). As the WHO/ISUP grading system has recently been proposed to replace the Fuhrman grading system, we further identified the grade of our cohort according to the International Society of Urological Pathology (WHO/ISUP) grading system.

Hypertension was defined as systolic blood pressure ≥ 140 mm Hg, diastolic blood pressure ≥ 90 mm Hg. Diabetes was defined as a fasting plasma glucose ≥ 126 mg/dL, 2-hour plasma glucose value ≥2 00 mg/dL after 75 g oral glucose tolerance test (OGTT). The pre-operative HbA1c data was also collected from DM patients. The tumor size of patients was classified based on SSIGN [3] and Leibovich [4] scores, namely, < 5 cm, 5–10 cm or > 10 cm. Progression-free survival was defined as the time from nephrectomy to distant metastasis or local recurrence.

### Measurements of SFA, VFA and RSFA

**Figure 4:**
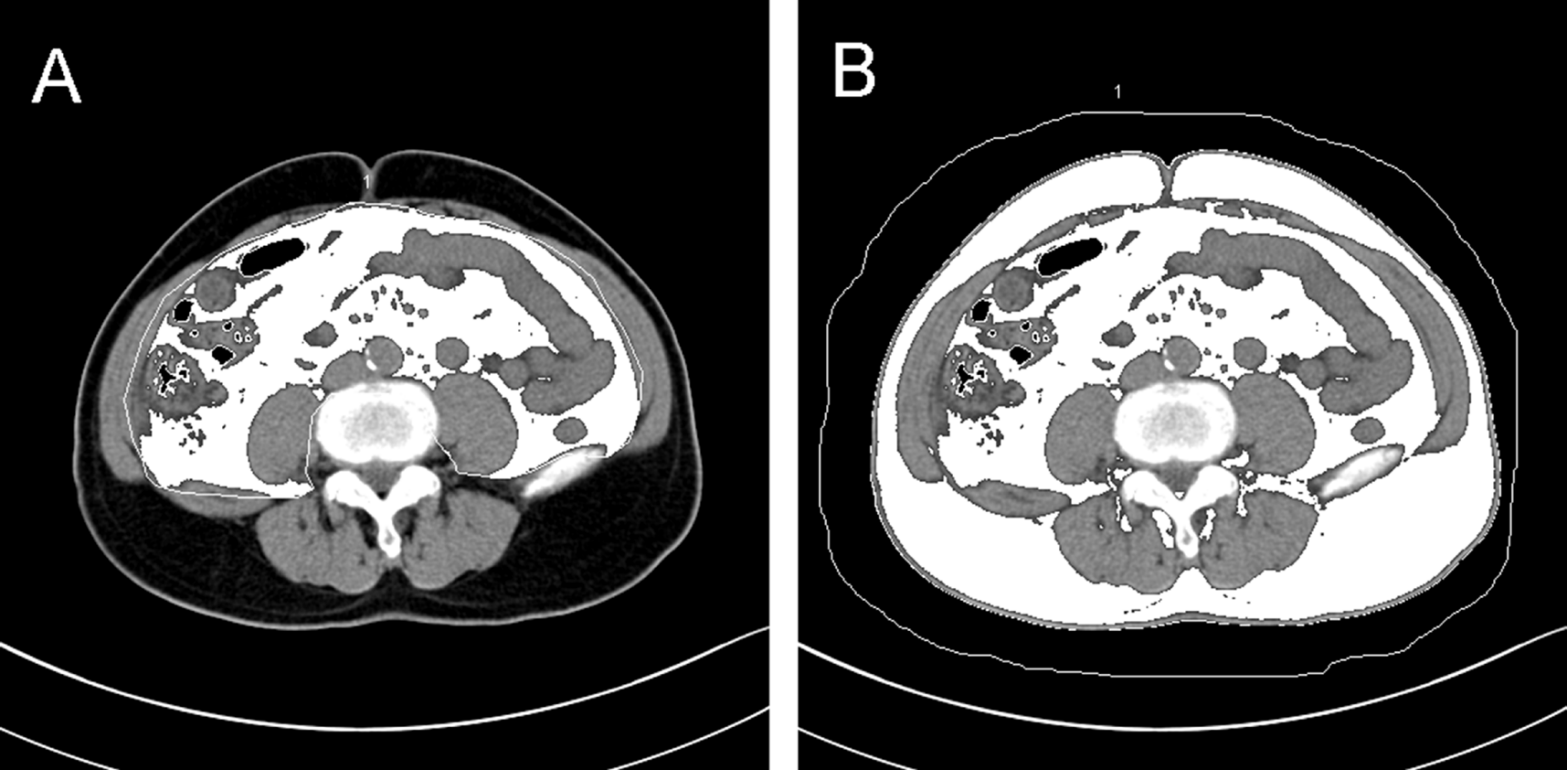
Representative axial CT images at the level of umbilicus showing (**A**) visceral fat area and (**B**) subcutaneous fat area plus visceral fat area.

**Figure 5:**
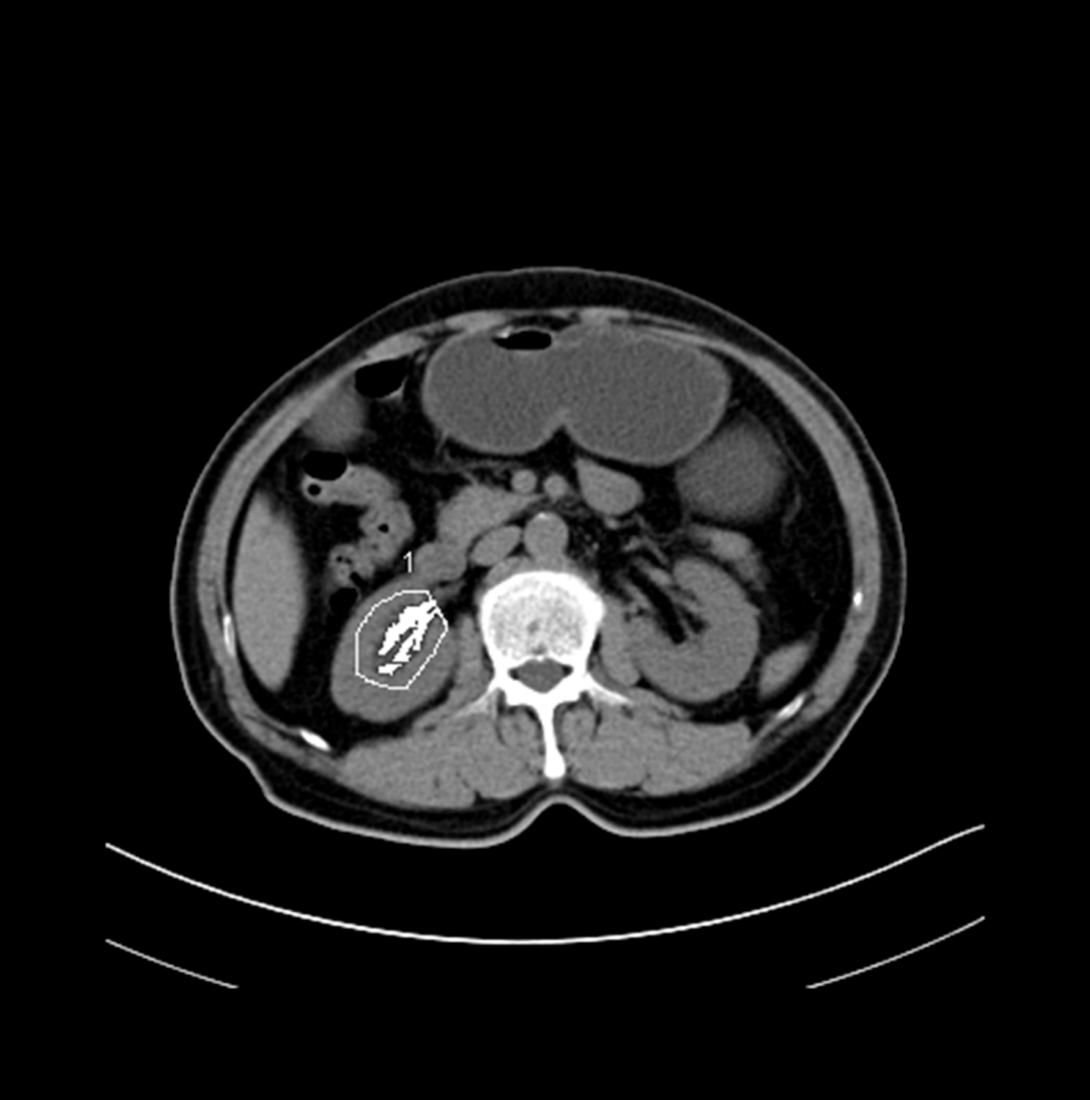
Representative axial CT image showing renal sinus fat area

Routine CT scans quantified the area of adipose tissue, including SFA, VFA and RSFA [8–15]. Abdominal CT scans (Somaris/7 CT 2012B, Siemens AG, Germany) were performed for all the enrolled patients before surgery. The contours of the abdominal muscular wall separating the visceral from the subcutaneous compartment at the level of umbilicus was manually outlined (Figure 4A–4B). Then, using an image display window width of –195 to –45 Hounsfield units, pixels containing fat were identified. The VFA and SFA values were then calculated with Syngo multimodality workplace, version VE31A (Siemens AG, Germany). Figure 5 shows the quantification of RSFA on the side of the lesion as described previously [15].

Measurements of the adipose tissue area were performed by one radiologist blinded to the clinical and pathological data. The sex-specific median value (123.11 cm^2^ for male; 80.92 for female) was used as the cut-off to distinguish patients with high and low VFA values [11]. Likewise, sex-specific median values of SFA (128.05 cm^2^ for male; 184.81 for female) were used to determine patients with high and low VFA. Since RSFA is potentially associated with gender and kidney side, the median sex- and side-specific median values (males: 1.28 cm^2^ and 0.91 cm^2^ for right and left kidneys; females: 0.59 cm^2^ and 0.46 cm^2^ for right and left kidneys) were used to distinguish high and low renal sinus fat accumulation.

### Statistical analysis

SPSS 22.0 (version 13; SPSS Inc., IL, USA) was used for all statistical analyses. We performed one to one propensity-score matched (PSM) analysis to reduce the effect of confounding factors and bias caused by different baseline distribution of demographic and clinicopathologic factors between patients with high or low RSFA.

Numerical variables were compared with *t*-test and categorical variables with chi-square test. Survival curves were plotted by the Kaplan-Meier method and assessed by the Log-rank test. Subsequently, Cox regression analysis was performed to identify potential prognostic factors for survival. Characteristics with *p* < 0.1 were further evaluated by multivariable Cox regression model. Then, the survival curves were used to analyze the differences between high and low RSFA groups in the PSM matched cohorts when all variables were comparable in the two groups. Previously, radical and partial nephrectomies showed similar survival outcomes for localized ccRCC [1]. Therefore, patients that underwent partial nephrectomy in this study were all identified as localized cases (81 as T1 stage, 1 as T2 stage) and the nephrectomy types were not enrolled for Cox and propensity-score matching analyses. We used both Fuhrman and WHO/ISUP grading systems to evaluate the grade of our cohort. Both grading systems were analyzed by univariable and multivariable Cox regression model.

All the independent predictive factors were organized into a prognostic nomogram. The nomogram construction and calibration was performed with the R software 3.3.3. The Harrell’s concordance index (c-index) was determined for the Leibovich score system and the new nomogram to assess prognostic accuracy. A *p* < 0.05 was considered statistically significant.

## Abbreviations

RCC: renal cell carcinoma
ccRCC: clear-cell renal cell carcinoma
MS: metabolic syndrome
VFA: visceral fat area
SFA: subcutaneous fat area
RSFA: renal sinus fat area
BMI: body mass index
PSM: propensity-score matched
PFS: progression-free survival
ISUP: International Society of Urological Pathology
